# Comparisons of aerosol backscatter using satellite and ground lidars: implications for calibrating and validating spaceborne lidar

**DOI:** 10.1038/srep42337

**Published:** 2017-02-15

**Authors:** Gary Gimmestad, Haviland Forrister, Tomas Grigas, Colin O’Dowd

**Affiliations:** 1 School of Physics and Centre for Climate and Air Pollution Studies, Ryan Institute, National University of Ireland Galway, Galway, Ireland; 2 School of Earth and Atmospheric Sciences, Georgia Institute of Technology, Atlanta, Georgia 30332, USA

## Abstract

The Cloud-Aerosol Lidar with Orthogonal Polarization (CALIOP) instrument on the polar orbiter Cloud-Aerosol Lidar and Infrared Pathfinder Satellite Observation (CALIPSO) is an elastic backscatter lidar that produces a global uniformly-calibrated aerosol data set. Several Calibration/Validation (Cal/Val) studies for CALIOP conducted with ground-based lidars and CALIOP data showed large aerosol profile disagreements, both random and systematic. In an attempt to better understand these problems, we undertook a series of ground-based lidar measurements in Atlanta, Georgia, which did not provide better agreement with CALIOP data than the earlier efforts, but rather prompted us to investigate the statistical limitations of such comparisons. Meaningful Cal/Val requires intercomparison data sets with small enough uncertainties to provide a check on the maximum expected calibration error. For CALIOP total attenuated backscatter, reducing the noise to the required level requires averaging profiles along the ground track for distances of at least 1,500 km. Representative comparison profiles often cannot be acquired with ground-based lidars because spatial aerosol inhomogeneities introduce systematic error into the averages. These conclusions have implications for future satellite lidar Cal/Val efforts, because planned satellite lidars measuring aerosol backscatter, wind vector, and CO_2_ concentration profiles may all produce data requiring considerable along-track averaging for meaningful Cal/Val.

When new spaceborne remote sensing instruments are launched, calibration-validation campaigns (commonly known as Cal/Val) are typically mounted. The purpose of Cal/Val is to characterize an instrument’s performance to ensure that it meets science needs. Calibration involves both pre-launch and post-launch measurements; here, we are concerned with the on-orbit phase of Cal/Val^1^. Validation often includes the process of assessing the overall confidence in a reported measurement result. This confidence is expressed as the estimated standard deviation assigned to a reported result, and it is often derived from intercomparisons between satellite measurements and independent reference measurements. It is this statistical definition that we address in this paper.

Lidar (light detection and ranging) instruments employ pulsed lasers with co-located receiver telescopes and fast electronics to record range-resolved profiles of backscattered light from atmospheric aerosols and molecules. The CALIOP (Cloud-Aerosol Lidar with Orthogonal Polarization) instrument on the polar orbiter CALIPSO (Cloud-Aerosol Lidar and Infrared Pathfinder Satellite Observation) is an elastic backscatter lidar, launched in April 2006, which produces a global uniformly-calibrated data sets of atmospheric backscatter profiles for several purposes, including improved predictive capability for air quality^2^. CALIOP initially produced Level 1 and Level 2 data products that included vertical profiles of attenuated backscatter in which the signal from any given altitude is attenuated by the atmosphere above that altitude, because both transmitted and scattered light must pass through that portion of the atmosphere. In several Cal/Val studies, the CALIOP Level 1 data were compared with data from ground-based lidars and estimates of calibration biases were reported with standard deviations^3^,^4^,^5^. The comparisons showed large random uncertainties as well as systematic errors in the boundary layer.

Since November 2010, an expedited CALIOP Level 1.5 near-real-time product, usually provided between 6 and 30 h after downlink, has been made available by NASA for purposes of operational forecasting^6^. Under the MACC (Monitoring Atmospheric Composition and Climate) project, the Level 1.5 total attenuated backscatter profile was assessed for assimilation into the ECMWF (European Centre for Medium-Range Weather Forecasts) global forecasting model IFS-MOZART (Integrated Forecast System coupled to the Model for OZone And Related chemical Tracers). That assessment used a statistical appraisal of the agreement between CALIOP data and archived EARLINET (European Aerosol Research Lidar Network^7^) profiles using three measures of comparison: the mean bias, the factor of exceedance, and the correlation coefficient, along with scatterplots. The scatterplots showed ground and satellite aerosol profile differences as large as ±50%^8^.

In an attempt to better understand both the large random differences and the systematic differences between satellite and ground-based lidar measurements in previous studies, we undertook a series of ground-based lidar measurements at Agnes Scott College (ASC) in Atlanta, Georgia, which is only 12 km from the nighttime CALIOP ground track at nearest approach. For each episode, we acquired the corresponding CALIOP Level 1.5 total attenuated backscatter profile and compared it with our ground-based lidar data. Our measurements did not provide better agreement with CALIOP data than our previous statistical study using Level 1.5 data or the earlier ground-based efforts using Level 1 data. The significance of the Atlanta study is that it prompted us to re-examine the particular difficulties and challenges presented by Cal/Val for satellite lidars, and how those challenges were addressed by the methodologies of other research teams. Through this process, we arrived at the scientific question that we addressing:

*What are the statistical considerations for planning a successful Cal/Val campaign for a spaceborne lidar, and what characteristics of the atmosphere must be considered*?

The answers to this question may have significant implications for the planning of future satellite lidar Cal/Val campaigns.

## Results

### Atlanta Measurements

Because the ASC lidar project led us to formulate the scientific question but did not directly shed new light on it, the project is described under Methods and in the Supplementary Information, with only a few comments on the two types of errors presented here: (1) Random errors. The random noise in the CALIOP profiles is much higher than the ASC profiles due to their much longer measurement range and shorter integration times, and the daytime noise level is higher than nighttime due to the statistical fluctuations associated with solar background light. The subjective agreement between the CALIOP and ASC profiles in the aerosol-free atmosphere above 10 km is good in all cases in the sense that the CALIOP data excursions are roughly centered around the ASC profiles. This finding is not surprising, because the ASC profile conversion requires a retrieval algorithm that normalizes the total backscatter profile to the molecular backscatter profile, calculated from radiosonde air density, at altitudes above the aerosols. Good agreement only means that radiosonde data recorded within a few hours at a nearby site are representative of air density observed by CALIOP. (2) Systematic errors. In the boundary layer and a few kilometers above it, the disagreement between the ASC lidar and CALIOP is often systematic. The two profiles have differences well outside of CALIOP random noise excursions in about half of the episodes. Occasionally, the top of the boundary layer is not even apparent in the CALIOP data. No seasonal or day versus night trends in the disagreement were observed in this limited data set, despite a much closer CALIOP-to-ASC offset distance at night and the lack of solar background noise for night episodes. The best agreement occurs when aerosol backscatter is lowest, but once again this is not surprising because those profiles are dominated by molecular backscatter. Unfortunately, all of our daytime summer overpasses were dominated by clouds, so we could not compare profiles with high aerosol optical depth on a warm summer day with CALIOP data. Our comparisons in Fig. S2 do not show appreciably better agreement than in the previous statistical study^8^ or in Level 1 data comparisons at EARLINET ground lidar stations. This finding suggested to us that there are some underlying problems in ground lidar Cal/Val methods, and prompted us to look into statistical limitations due to the CALIOP random noise.

### Statistical considerations

**Figure 1:**
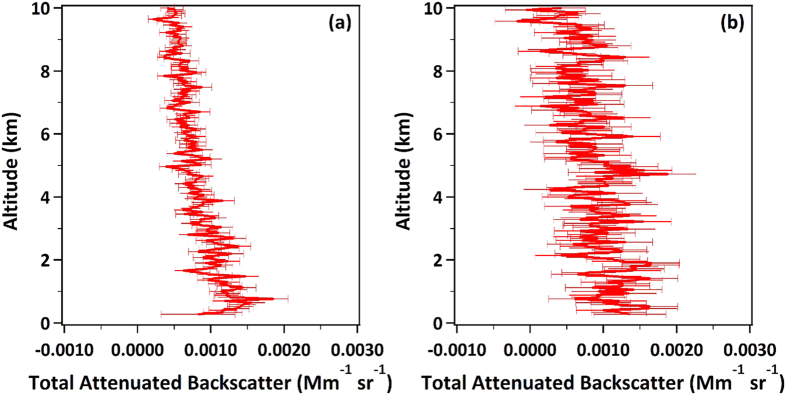
Mean and uncertainty values of CALIOP data in the 0–10 km altitude range. CALIOP Level 1.5 total attenuated backscatter mean (solid red line) and uncertainty (error bars) are shown for closest approach on (**a**) 13 November 2013, a typical nighttime data set, and (**b**) 14 January 2014, a typical daytime data set. The along-track averaging distance is 20 km.

To address the large random errors, it is instructive to calculate the amount of averaging that would be required to reduce the rms noise of the CALIOP profiles to the 2% level reported for an airborne Cal/Val campaign^9^. The estimated maximum bias in the CALIOP calibration is 5%^10^, so 2% uncertainty is small enough to enable a check on that value. In our study, we used Level 1.5 total attenuated backscatter profiles, which have an along-track resolution of 20 km. Level 1.5 mean and uncertainty profiles for CALIOP’s closest approach to Atlanta on 13 November 2013 (nighttime) and 14 January 2014 (daytime) are shown in Fig. 1. Nighttime uncertainties are primarily due to signal noise (statistical fluctuations in the arrival rate of photons) and are a function of the signal level and hence, altitude. Daytime uncertainties are much larger and are nearly constant with altitude because they are dominated by solar background noise, which does not change with altitude. For the purpose of illustration, we will use an altitude of 5 km, which is a mid-range altitude in our study as well as in other reported CALIOP Cal/Val studies. The uncertainties as percentages of the mean at 5 km altitude are shown in Table 1 for all of our episodes, along with their average values. These values were averaged over a 1-km altitude region centered on 5 km. Notably, a 20-km along-track average has 17% uncertainty at night and 49% during day. These values are larger than the maximum 5% error that CALIOP Cal/Val is intended to check, necessitating more averaging in order to reduce the uncertainty.

**Figure 2:**
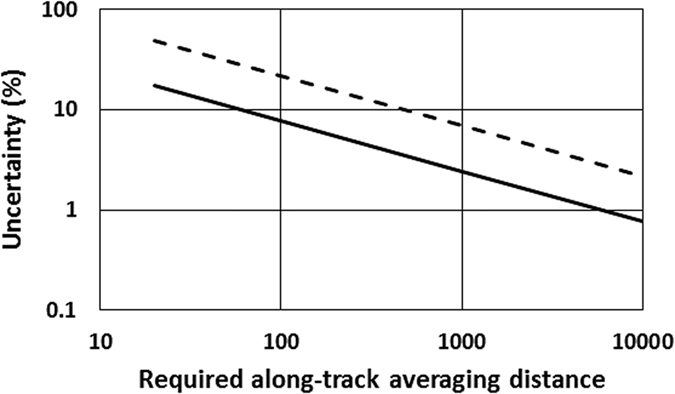
CALIOP uncertainty at 5 km altitude. The along-track averaging distance required to achieve a given uncertainty is shown for day (dashed line) and night (solid line).

To achieve 2% uncertainty for CALIOP Cal/Val for our case study, the noise level in the final averaged CALIOP profile must decrease by a factor of 8.6 on average, for nighttime. If this decrease in noise is proportional to the square root of the number of samples, 74 of the 20-km Level 1.5 nighttime profiles must be averaged together. This number of profiles corresponds to 1,480 km of along-track averaging. The daytime requirements are 590 profiles and 11,800 km. These calculations are for the best case scenario: a noise decrease proportional to the square root of the sample number is only expected for independent Gaussian samples with stationary statistics. Unless CALIOP and the comparison instrument measure the atmospheric aerosol profiles on the same optical paths, the statistics may not be stationary. The percentage uncertainties calculated here strictly pertain only to our Atlanta overpasses, but in general terms, this case study indicates that achieving 2% uncertainty at 5 km altitude in CALIOP Cal/Val studies requires along-track averaging of thousands of km during nighttime and tens of thousands of km during daytime. The required along-track averaging distances are shown in Fig. 2 for a range of desired uncertainties.

In our experimantal study, we used 100-km averages of CALIOP profiles, as did Grigas *et al*.^8^. The protocol employed by previous Cal/Val studies with Level 1 data used 5-km averaged profiles, which would require the acquisition of such profiles on nearly 300 nighttime overpasses in order to achieve 2% uncertainty. Acquiring 300 Cal/Val profiles in the same location is probably beyond the means of any one lidar station. In addition, the same aerosol backscatter profile would almost certainly not be observed by CALIOP and the ground lidar over the course of 300 profiles due to aerosol inhomogeneity. Averaging over long along-track distances reduces *random* error, but it may well introduce *systematic* error. This problem was demonstrated by our Atlanta study and by other CALIOP Cal/Val studies: the typical protocol of using ground track offsets <100 km does not guarantee that the CALIOP and ground lidar will see the same aerosol backscatter profile, especially in the boundary layer. Even with our relatively small 12-km offset, we frequently saw obvious differences in the boundary layer, as shown in Fig. S2. These results suggest that ground lidars often cannot provide Cal/Val intercomparison data that is useful for quantifying the CALIOP calibration error at its expected value of a few percent.

## Discussion

In our experimental study, we recorded and analysed ground lidar data for comparison with CALIOP profiles, and found large random errors as well as frequent systematic disagreements below the free troposphere. These phenomena had been reported in several previous studies and studied in terms of the representativeness of ground lidar data from a given site for comparison with CALIOP. We then investigated the statistics of Cal/Val schemes to elucidate the circumstances under which quantitative Cal/Val of a satellite lidar is possible with ground lidars. We found that about 1,500 km of along-track averaging of CALIOP profiles is needed to achieve 2% uncertainty, for nighttime comparisons. None of the reported ground lidar Cal/Val efforts achieved such a small uncertainty.

**Figure 3:**
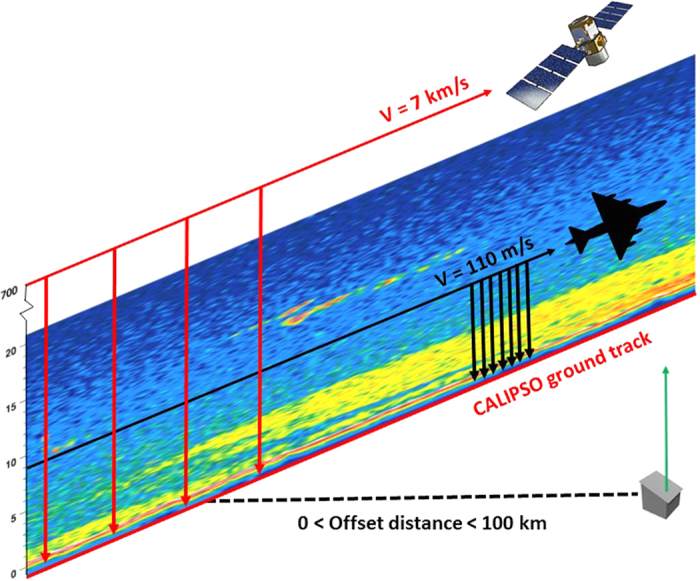
Cal/Val Methodologies. The nadir-viewing spaceborne and airborne lidars spatially averaged aerosol backscatter profiles in the same sheet of the atmosphere with a time offset of no more than 45 min. The zenith-viewing ground lidars time-averaged profiles at one location, offset by as much as 100 km from the CALIPSO ground track. The arrows represent individual laser pulses and lidar viewing directions. Distances are not to scale. (Source of the sattelite image in the figure: NASA A-Train figure^13^).

The measurement methodologies of CALIOP, ground, and airborne lidars are distinctly different, as illustrated in Fig. 3. CALIOP averages quick measurements (moving at ~7 km/s along the ground track) of vertical atmospheric sheets, looking downward. In contrast, ground lidars average over longer time periods at a single location (usually urban), looking upward, using Raman or elastic backscatter lidars that require retrieval algorithms to calculate downward-looking total attenuated backscatter. The airborne measurements used a nadir-viewing HSRL (High Spectral Resolution Lidar) and flew under the CALIPSO satellite. The airborne instrument probed the same vertical atmospheric plane as CALIOP, allowing both instruments to average the same aerosol backscatter profiles. Therefore, the long averaging tracks did not introduce systematic errors, despite aerosol inhomogeneities, and distortions caused by converting zenith-viewing lidar signals to nadir-viewing were avoided. The CALIOP and HSRL measurements were not simultaneous, but Rogers *et al*.^9^ showed that HSRL profiles displaced in time by no more than 45 minutes from CALIOP did not show significant differences. At the airplane’s nominal ground speed of 110 m/s, 45 minutes corresponds to about 300 km of along-track averaging, which is significantly more than any of the ground-based efforts. More importantly, the lack of artefacts allows for multiple-flight averaging. Combined, these similarities between the CALIOP and airborne HSRL measurement methodologies allowed the airborne researchers to average CALIOP noise down to a few percent and conduct quantitative Cal/Val.

Our findings have implications for future satellite lidar Cal/Val efforts. Lidar data from satellites will always have greater noise than ground lidars, due to their great speed and orbital distance. To plan successful Cal/Val campaigns, the lidar community will require: (1) an estimate of the noise level in profiles generated by the satellite sensor; and (2) an estimate of the calibration uncertainty that the Cal/Val effort is intended to check. Researchers can then calculate the number of satellite profiles that must be averaged to provide quantitative Cal/Val, as well as the practicality of proposed Cal/Val campaigns in terms of the data acquisition time. For ground lidar measurements, researchers must also consider what is known about the homogeneity of the parameter of interest (aerosol backscatter, wind vector, CO_2_ concentration, etc.) at the lidar site to assess whether averaging multiple profiles can be accomplished without introducing systematic errors.

## Methods

### Instrumentation

The ground lidar data were acquired with a micropulse lidar developed by the Georgia Tech Research Institute (GTRI) and Agnes Scott College (ASC) under a grant from the National Science Foundation to develop an educational lidar^11^. The ASC lidar is located in Atlanta, Georgia. The lidar employs a 2500 Hz doubled Nd:YLF laser transmitting 14 μJ per pulse at 523.5 nm. To accommodate a wide range of altitudes, the lidar receiver has short-range and long-range channels that receive 9% and 91% of the light, respectively. The short range channel achieves complete crossover at 500 m. The ASC lidar is a simple elastic backscatter lidar, which records an uncalibrated signal in two receiver channels, digitized at 15-m altitude intervals and time-averaged as desired. More detail is given in Forrister *et al*.^12^. For the work reported here, each data file was the average from 80,000 laser pulses and the files were recorded continuously for two hours.

The CALIOP lidar is a nadir-viewing 532-nm elastic backscatter lidar. Its pulse repetition frequency (~20 Hz) determines the laser pulse spacing of 333 m along the ground track, or 3 pulses/km. The CALIPSO orbit is inclined such that the ground tracks repeat every sixteen days. In Atlanta, the closest ground tracks occur first during daytime, with an average offset distance of 80 km, and two days later during nighttime, with an average offset of 12 km.

The Level 1.5 data product used in our comparisons is a near-real-time product that was developed by NASA for potential assimilation into an aerosol forecast model. The Level 1.5 total attenuated backscatter profile is used in our comparisons. The term “total” indicates the sum of the parallel and cross polarization signals for molecular and aerosol backscatter at each range interval. CALIOP total attenuated backscatter data is calibrated and reported in absolute units (km^−1^ sr^−1^). The Level 1.5 data files also include the predominant aerosol type, which is typically Urban or Clear Continental near Atlanta, Georgia.

### Measurement Procedure

**Figure 4:**
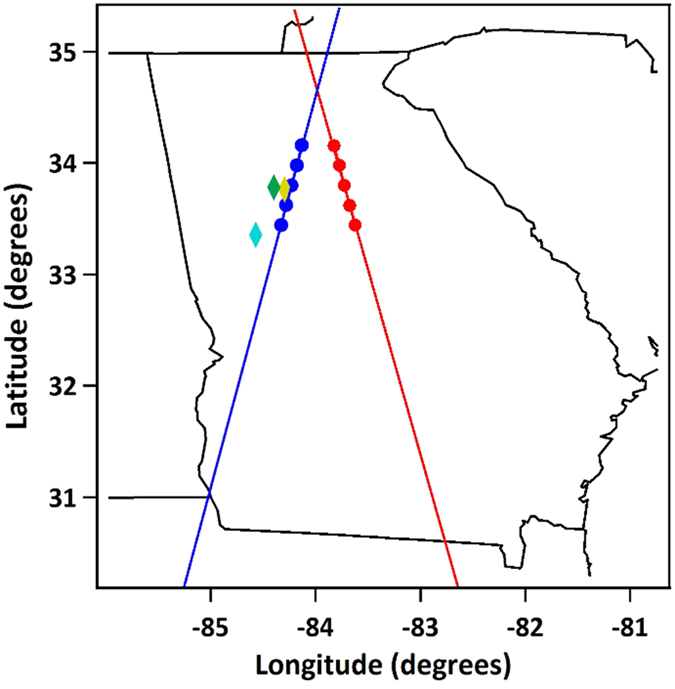
Instrument and ground track locations. The ASC lidar (gold diamond) and AERONET (green diamond) are in Atlanta, GA; the radiosonde launch site (blue diamond) is in Peachtree City, GA; and the CALIOP ground tracks are shown for nighttime (blue line) and daytime (red line). The blue and red dots indicate the locations of the five closest-approach Level 1.5 data files that were averaged.

The lidar ASC lidar is 8.3 km due east of the AERONET sun photometer at GTRI; both are located in Atlanta, Georgia. The National Weather Service (NWS) balloon launch site for upper air data is 48.5 km SSW of the sun photometer. A map designating instrument locations as well as nominal CALIOP ground tracks is shown in Fig. 4; details are listed in Forrister *et al*.^12^.

We initially believed that the small offset distance between ASC and the nighttime ground track made the ASC lidar a good choice for this comparison study. Also, the ASC lidar, with its low crossover altitude, was designed for studies in the boundary layer as well as higher altitudes. Our data acquisition episodes included a variety of wind directions, causing CALIOP and the ASC lidar to not always measure the same air mass. However, measurement biases did not appear to be correlated with wind direction or speed.

Additional details on the ASC lidar data acquisition and data analysis procedures are presented in the Supplementary Information.

## Additional Information

**How to cite this article:** Gimmestad, G. *et al*. Comparisons of aerosol backscatter using satellite and ground lidars: implications for calibrating and validating spaceborne lidar. *Sci. Rep.* **7**, 42337; doi: 10.1038/srep42337 (2017).

**Publisher's note:** Springer Nature remains neutral with regard to jurisdictional claims in published maps and institutional affiliations.

## Supplementary Material

Supplementary Information

## Figures and Tables

**Table 1:** CALIOP uncertainties as percentages of the mean value at 5 km altitude.

Nighttime		Daytime	
22 Jun 2013	17.2%	10 Oct 2013	56.7%
12 Oct 2013	16.8%	11 Nov 2013	52.3%
13 Nov 2013	17.5%	14 Jan 2014	36.9%
16 Jan 2014	17.2%	30 Jan 2014	48.6%
2 Feb 2014	17.1%		
Average	17.2%	Average	48.6%
