# Supplementary material for: Comparisons of aerosol backscatter using satellite and ground lidars: implications for calibrating and validating spaceborne lidar
Source: Sci Rep. 2017 Feb 15;7:42337. doi: 10.1038/srep42337 (PMC5309828; doi:10.1038/srep42337)
Supplement: Supplementary Information [file srep42337-s1.pdf]

Comparisons of aerosol backscatter using satellite and ground lidars: implications for calibrating and validating spaceborne lidar

Gary Gimmestad, Haviland Forrister, Tomas Grigas, and Colin O'Dowd

Data acquisition

The ASC (Agnes Scott College) lidar was operated during CALIOP (Cloud-Aerosol Lidar with Orthogonal Polarization) overpasses every time the sky was either clear or had few clouds, typically from one hour before the overpass to one hour after. Data were acquired during night and day, yielding a total of nine data acquisition episodes from June 2013 to February 2014. Because clear sky conditions were desired, time intervals when clouds appeared were not included in the data averaging, which occasionally decreased ASC temporal averaging from 2 hours to 75-95 minutes. Supplementary Table S1 contains a list of the dates and times of each episode, as well as information about cloud removal. To determine the molecular density of air as a function of altitude, we used pressure and temperature data from local radiosonde profiles at 00:00 UTC for the daytime data and 12:00 UTC for the nighttime data. AERONET (Aerosol Robotic Network) aerosol optical depth (AOD) and absorption Angstrom exponent data (AAE) were used to check the conversion of the ground lidar signal into nadir-viewing geometry, but were only available during daytime. Therefore, nighttime data conversion used the earliest AERONET data for the following day. The total ozone optical depth for Atlanta was used to calculate ozone absorption using the World Ozone and Ultraviolet Radiation Data Centre[1].

Table S1. ASC lidar data acquisition episodes

Date	Time of Day	Closest Approach (UTC)	Time Interval (UTC)	Cloud Removed?
22 Jun 2013	Night	07:42	06:15 – 07:30	Yes
10 Oct 2013	Day	19:07	15:15 – 16:50	Yes
12 Oct 2013	Night	07:42	06:19 – 08:08	No
11 Nov 2013	Day	19:07	18:07 – 20:09	No
13 Nov 2013	Night	07:42	06:55 – 08:42	No
14 Jan 2014	Day	19:07	19:09 – 21:07	No
16 Jan 2014	Night	07:42	06:43 – 08:39	No
30 Jan 2014	Day	19:07	17:49 – 19:50	No
02 Feb 2014	Night	07:42	06:18 – 08:43	No

Data Analysis

CALIOP Level 1.5 data is reported as total attenuated backscatter in units of km^{-1} sr^{-1}, viewing from the top of the atmosphere downward. Each profile contains an average of 60 individual attenuated backscatter profiles from the Level 1 data product merged with cloud-and-aerosol-layer detection from the Level 2 data product. As such, Level 1.5 data has its own unique spatial averaging and resolutions. The averaged profile, which covers a 20-km

horizontal distance along the ground track, is reported at 60-m altitude intervals from -500 to 20,000 m mean sea level (MSL)[2].

ASC lidar data is exported in terms of range-corrected, background-subtracted digitizer counts for each 80,000-pulse average in two receiver channels: short-range (SR) and long-range (LR). The SR channel has better signal-to-noise ratio (SNR) up to 2-2.5 km while the LR channel is better above 2-2.5 km. Both channels contain vertical profiles to 30 km in 15-m altitude increments. To obtain the best SNR over the entire vertical profile, we merged the SR and LR data at an altitude where both channels had achieved full crossover and had high SNR.

In elastic backscatter lidar measurements, the signal from any altitude is attenuated by the atmosphere between that altitude and the lidar, due to molecular scattering as well as aerosol scattering and absorption. For this reason, nadir-viewing and zenith-viewing lidars observing the same atmosphere are not directly comparable, so the zenith-viewing ground lidar data must be converted into the downward view of CALIOP. In some circumstances, this conversion process distorts the profile[3]. To convert our profiles, we used the equations described by Grigas et al.[4] to calculate the profiles for backscatter $\beta(z)$ and extinction $\alpha(z)$ of both the molecular (*mol*) and aerosol (*aer*) data.

Although we used similar methods, our procedure for obtaining the profiles of $\beta_{mol}(z)$, $\beta_{aer}(z)$, $\alpha_{mol}(z)$, and $\alpha_{aer}(z)$ varied slightly from Grigas et al.[4] Elastic backscatter lidars, such as the ASC lidar, require a Klett algorithm, constrained by AOD values, to separate $\beta_{aer}(z)$[5]. The Klett algorithm requires $\alpha_{mol}(z)$ and $\beta_{mol}(z)$ as inputs, which we computed using molecular number density from local radiosonde data and cross-sections for extinction and scattering at 523.5 nm. The molecular profiles were interpolated into the same altitude intervals as the ASC data and were converted to 532 nm.

The Klett Algorithm is a recurrence relation that starts at a highest altitude and works downward to the lowest altitude at which the lidar has achieved complete crossover (500 m in our case). At the highest altitude, the value of β_{aer} must be known, so it is convenient to pick an aerosol-free altitude where the value of β_{aer} is zero. To find the altitudes where the atmosphere was aerosol-free, the averaged range-corrected ASC signal was plotted along with β_{mol} scaled to match the lidar signal at the highest altitudes, as shown in Supplementary Fig. S1. The altitude at which the two curves merged was taken as the lowest aerosol-free altitude.

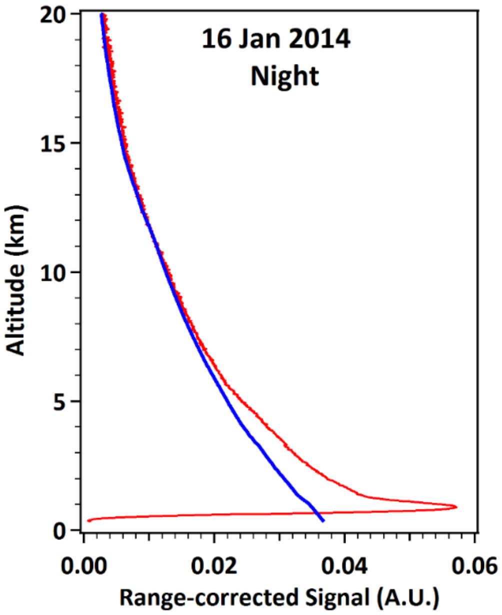

Figure S1**.** An example fit of the molecular backscatter (blue) to the ASC lidar signal (red).

The Klett algorithm also requires S_a to be known. For polluted urban aerosol, CALIOP uses S_a = 70 sr, so this value was used in all of our retrievals except when the aerosol level was very low, in which case we assumed clean continental aerosol (S_a = 35 sr)[6]. After retrieving the $\beta_{aer}(z)$ profile for each episode, we found $\alpha_{aer}(z)$ according to Eq. 6 from Grigas et al.[4] To validate the assumed S_a, we extrapolated our $\alpha_{aer}(z)$ profile down to the surface and then integrated it from the surface to the top. The resulting lidar AOD was then compared to the value measured at the Georgia Tech AERONET station. The AERONET values are displaced in time from the nighttime lidar measurements, so they may not be very representative for those data sets. The comparisons are shown in Supplementary Table S2. On all occasions but two (14 and 16 January 2014), the S_a value we used matched the reported CALIOP aerosol type. 16 January 2014 was the only nighttime data set with very high boundary layer signals, so we used a polluted air value. 14 January 2014 had an active boundary layer that CALIOP did not see, so we used the polluted value even though CALIOP saw clear air. All of the other S_a values match CALIOP's reported aerosol class.

Once $\beta_{aer}(z)$ and $\alpha_{aer}(z)$ were determined for the ASC lidar's altitude range, the coefficients in the aerosol-free region were set to zero while the coefficients at other altitudes were translated from 523.5nm to 532 nm using the AAE measured at GTRI's AERONET station. Total backscatter was calculated using $\beta_{mol}(z)$ and $\beta_{aer}(z)$. Total extinction was calculated using $\alpha_{mol}(z)$ and $\alpha_{aer}(z)$, as well as ozone extinction. The ozone number density is included in the Level 1.5 data products, but only up to 20 km, which includes approximately one-half of the total ozone. For this reason, ozone data in Dobson units was retrieved for each episode from the World Ozone and Ultraviolet Radiation Data Centre. Dobson units are column-integrated, and one unit corresponds to 2.69 x 10^{20} molecules/m^2. The ozone cross section at 532 nm is 2.7 x 10^{-25} m^2/molecule[7], yielding an optical depth per Dobson unit of 7.3 x 10^{-5}.

The two-way transmittance was calculated using the total extinction values and then multiplied by the total backscatter to find the total attenuated backscatter for a nadir-viewing lidar. We smoothed our values to 60-m resolution using a moving-average filter. Both the ASC and CALIOP total attenuated backscatter data were expressed in units of Mm^{-1} sr^{-1}. The CALIOP Level 1.5 data were averaged spatially using the five closest 20-km overpass data sets in order to improve their low SNR. The profiles were then plotted on the same graph for each episode.

Table S2. AOD comparisons

Date	Time of Day	Time Interval (UTC)	S_a	Lidar AOD	AERONET Time (UTC)	AERONET AOD
22 Jun 2013	Night	0615 – 0730	35	.1522	1120	.1456
10 Oct 2013	Day	1515 – 1650	70	.0907	1910	.0791
12 Oct 2013	Night	0619 - 0808	35	.0474	1220	.0374
11 Nov 2013	Day	1807 – 2009	35	.0456	1910	.0458
13 Nov 2013	Night	0655 – 0842	35	.0063	1250	.0291
14 Jan 2014	Day	1909 – 2107	70	.0511	1900	.0957
16 Jan 2014	Night	0643 – 0839	70	.0398	1350	.0666
30 Jan 2014	Day	1749 – 1950	70	.0375	1900	.0583
02 Feb 2014	Night	0618 – 0843	35	.0035	1320	.0416

Results

Supplementary Fig. S2 shows our comparisons of the nadir-viewing attenuated backscatter profiles converted from our ASC lidar data with the corresponding CALIOP profiles.

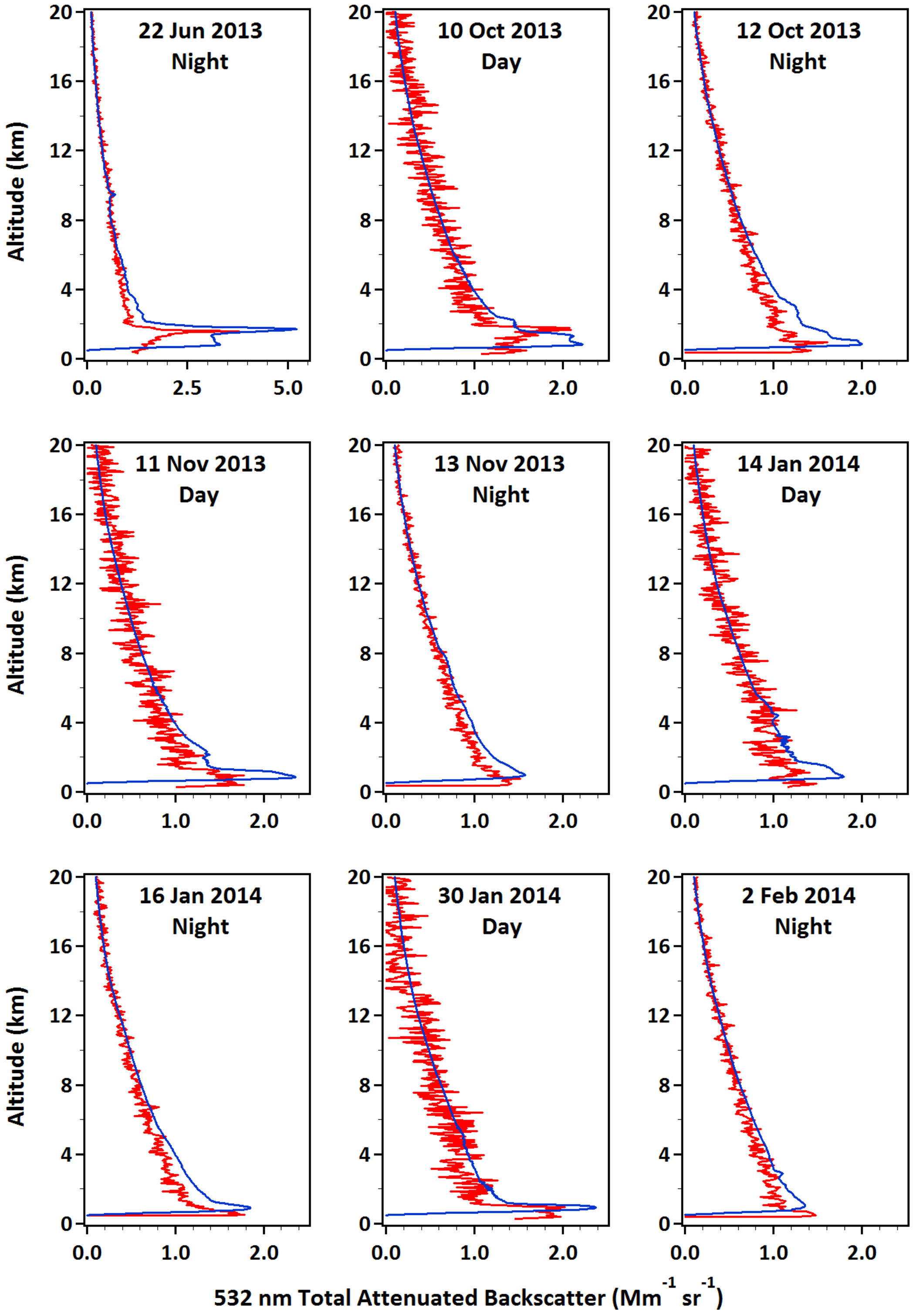

Figure S2. Comparisons of lidar profiles. Mean 532-nm attenuated backscatter profiles are shown for CALIOP (red) and the ASC lidar (blue).

References

1. World Ozone and Ultraviolet Radiation Data Centre. Available at: http://woudc.org. (Accessed: December 2016).

2. Powell, K. et al. CALIPSO Data Products Catalog, Release 3.6 (2013).

3. Ansmann, A. Ground-truth aerosol observations: can Klett solutions obtained from Ground and space be equal for the same aerosol case? *Applied Optics, 45,* 3367 – 3371 (2006).

4. Grigas, T., et al. On the performance of CALIOP near-real-time backscatter products compared to EARLINET. *Atmospheric Chemistry and Physics 15,* 12179 – 12191 (2015).

5. Klett, J.D. Stable analytical inversion solution for processing lidar returns, *Applied Optics, 20,* 211–220 (1981).

6. Omar, A.H., et al. The CALIPSO automated aerosol classification and lidar ratio selection algorithm. *Journal of Atmospheric and Oceanic Technology, 26,* 1994-2014, doi:10.1175/2009JTECHA1231.1 (2009).

7. Brion, J., et al. Absorption spectra measurements for the ozone molecule in the 350-830 nm region. *Journal of Atmospheric Chemistry 30,* 291-299 (1998).
